# Stabilization of endogenous Nrf2 by minocycline protects against Nlrp3-inflammasome induced diabetic nephropathy

**DOI:** 10.1038/srep34228

**Published:** 2016-10-10

**Authors:** Khurrum Shahzad, Fabian Bock, Moh’d Mohanad Al-Dabet, Ihsan Gadi, Sumra Nazir, Hongjie Wang, Shrey Kohli, Satish Ranjan, Peter R. Mertens, Peter P. Nawroth, Berend Isermann

**Affiliations:** 1 Institute of Clinical Chemistry and Pathobiochemistry, Otto-von-Guericke-University, 39120 Magdeburg, Germany; 2 University of Health Sciences, Khayaban-e-Jamia Punjab, 54600, Lahore, Pakistan; 3 Department of Medicine, Vanderbilt University Medical Center, 37232 Nashville, Tennessee, United States; 4 Department of Cardiology, Tongji Hospital, Tongji Medical College, Huazhong University of Science and Technology, Wuhan, China; 5 Clinic of Nephrology and Hypertension, Diabetes and Endocrinology, Otto-von-Guericke University Magdeburg, 39120 Magdeburg, Germany; 6 Department of Internal Medicine I and Clinical Chemistry, German Diabetes Center (DZD), University of Heidelberg, 69120 Heidelberg, Germany

## Abstract

While a plethora of studies support a therapeutic benefit of Nrf2 activation and ROS inhibition in diabetic nephropathy (dNP), the Nrf2 activator bardoxolone failed in clinical studies in type 2 diabetic patients due to cardiovascular side effects. Hence, alternative approaches to target Nrf2 are required. Intriguingly, the tetracycline antibiotic minocycline, which has been in clinical use for decades, has been shown to convey anti-inflammatory effects in diabetic patients and nephroprotection in rodent models of dNP. However, the mechanism underlying the nephroprotection remains unknown. Here we show that minocycline protects against dNP in mouse models of type 1 and type 2 diabetes, while caspase -3,-6,-7,-8 and -10 inhibition is insufficient, indicating a function of minocycline independent of apoptosis inhibition. Minocycline stabilizes endogenous Nrf2 in kidneys of db/db mice, thus dampening ROS-induced inflammasome activation in the kidney. Indeed, minocycline exerts antioxidant effects *in vitro* and *in vivo*, reducing glomerular markers of oxidative stress. Minocycline reduces ubiquitination of the redox-sensitive transcription factor Nrf2 and increases its protein levels. Accordingly, minocycline mediated Nlrp3 inflammasome inhibition and amelioration of dNP are abolished in diabetic Nrf2^−/−^ mice. Taken together, we uncover a new function of minocycline, which stabilizes the redox-sensitive transcription factor Nrf2, thus protecting from dNP.

Diabetic nephropathy (dNP) is the leading cause of end-stage renal disease and the strongest predictor of mortality in diabetic patients worldwide^1^. Knowledge about the molecular mechanisms of diabetic nephropathy progressed over recent years^2^,^3^,^4^. Blockade of the renin-angiotensin-aldosterone system (RAAS) by ACE inhibitors (ACEI) or angiotensin II receptor blockers (ARB) is still the mainstay of therapy, but only delays the initiation and progression of dNP in type 1 and type 2 diabetic patients. Efficient therapeutic approaches halting or even reversing progression of dNP are lacking, necessitating the search for new treatment strategies. One potential therapeutic target in dNP is the transcription factor Nrf2, which induces antioxidant and anti-inflammatory effects^5^,^6^. Nrf2 is a cellular sensor of oxidative stress and a nuclear factor controlling and coordinating expression of genes protecting against cellular damage provoked by excess ROS and other cellular stressors. Nrf2 belongs to the basic leucine zipper (bZIP) transcription factors and is repressed by Keap1 (Kelch-like erythroid cell-derived protein with CNC homology (ECH)-associated protein 1, also known as INrf2, inhibitor of Nrf2) and the Cul3/Rbx1 E3 ubiquitin ligase complex under physiologic conditions^7^. Under resting conditions Nrf2 interacts with and is ubiquitinated by Keap1 and the Cul3/Rbs1 E3 complex, targeting it for proteasomal degradation. Following appropriate stimulation, e.g. oxidative stress, inhibitors of Nrf2 including Keap1, Cul3, and Rbx1 E3 are exported from the nucleus and themselves ubiquitinated, allowing unhindered nuclear import and activity of Nrf2^7^. In parallel, Nrf2 is regulated by phosphorylation at specific serine or threonine residues^7^. To stimulate an antioxidant response Nrf2 dimerizes with members of the small Maf family or c-Jun and binds to antioxidant response elements (AREs) in the regulatory regions of cellular cytoprotective proteins, such as glutathione enzymes (glutathione reductase, microsomal glutathione S-transferase 2), heme metabolism enzymes (heme oxygenase 1, ferrochelatase) or antioxidant enzymes (manganese superoxide dismutase (MnSOD), peroxiredoxin 1, thioredoxin reductase 1)^8^,^9^. This allows Nrf2 to efficiently initiate a cytoprotective response and prevent increased oxidative stress and further cellular damage.

Pre-clinical studies using Nrf2 activators or Nrf2 deficient mice established that Nrf2 is a crucial endogenous modulator of ROS and protects from experimental dNP^10^,^11^. These pre-clinical data were supported by results form the BEAM trial, in which the Nrf2 activator bardoxolone improved kidney function in patients with chronic kidney disease and type 2 diabetes mellitus^12^. However, the subsequent and larger BEACON trial had to be prematurely terminated due to an increased rate of cardiovascular events, which may indicate off-target effects of bardoxolone^13^. This raises the question as to whether alternative approaches to restrict Nrf2 function are feasible, providing an effective yet safe therapeutic approach to dNP.

Minocycline, a tetracycline derivate with off-target effects such as modulation of mitochondrial function and apoptosis inhibition, has shown beneficial effects in patients with dNP and in pre-clinical kidney injury models^14^,^15^,^16^. A recent small study evaluating minocycline in patients with dNP revealed anti-inflammatory effects of minocycline in diabetic patients^17^. Furthermore, minocycline efficiently prevents dNP in type 1 diabetes models (streptozotocin) in mice and rats^18^,^19^. However, the mechanism underlying minocycline’s protective effect in dNP remains unknown. In renal ischemic-reperfusion injury models minocycline normalizes the Bax/Bcl-2 ratio, reduces cytochrome C release, and inhibits apoptosis in the kidney, suggesting nephroprotective effects of minocycline through inhibition of apoptosis^14^,^15^. An increased apoptosis rates–largely based on results using the non-specific TUNEL assay–has been repeatedly demonstrated in dNP^20^. Accordingly, minocycline mediated cytoprotection in dNP has been associated with reduced apoptosis. However, we recently demonstrated that caspase-3 mediated apoptosis is not required for dNP development in mice^21^. Based on these data we speculated that minocycline must convey protection from dNP independent of apoptosis.

While homozygous caspase-3 deficiency fails to protect from dNP in mice, both homozygous and hemizygous caspase-1 deficiency is protective, consistent with a causal role of caspase-1 dependent inflammasome activation in dNP^21^,^22^. Furthermore, we previously demonstrated that increased reactive oxygen species (ROS) generation causes renal inflammasome activation and dNP^22^. Beyond inhibition of apoptosis minocycline inhibits ROS-formation, pro-inflammatory cytokines, and IκBα degradation^23^,^24^–all pathways that are regulated by Nrf2 and of potential mechanistic relevance in dNP. Hence, we hypothesized that minocycline protects from dNP independent of apoptosis-inhibition by restricting excess ROS-generation potentially *via* a Nrf2-dependent mechanism, thus preventing inflammasome activation in dNP.

## Results

### Minocycline, but not a pan-caspase inhibitor, ameliorates dNP

**Figure 1:**
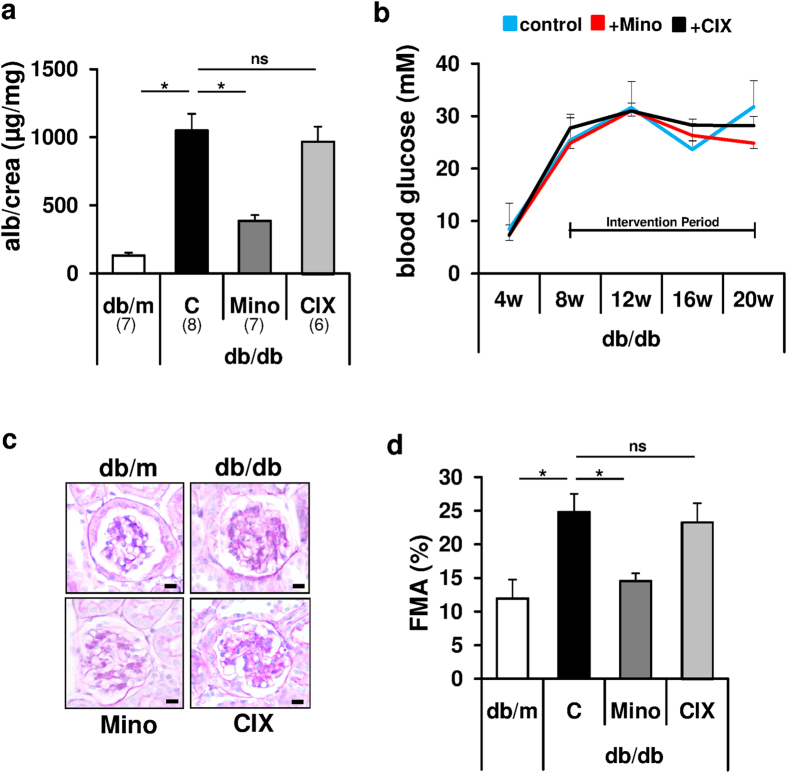
Minocycline, but not the pan-caspase inhibitor CIX, protects from diabetic nephropathy in db/db mice. Albuminuria (**a**) and extracellular matrix accumulation, as reflected by the fractional mesangial area (FMA, **c,d**) are decreased in minocycline (Mino) receiving db/db mice as compared to PBS-receiving db/db control mice (C, db/db). Application of CIX (+CIX, targeting caspases-3, -6, -7, -8 and -10) has no impact on these markers in db/db mice. Blood glucose levels are comparable among experimental groups (**b**). All treatment strategies were initiated at age 8 weeks and continued for 12 weeks. Mean values ± SEM (**a,b,d**). Representative PAS-stained glomeruli (c, size bar: 20 μm); *P < 0.05, ns: non-significant (**a,b,d**: ANOVA). Number of mice in each group is shown in parentheses in (**a**).

To gain insight into the mechanism underlying the nephroprotective effect of minocycline in dNP we compared the efficacy of minocycline (5 mg/kg, intraperitoneal, i.p.) with that of the caspase-inhibitor CIX (inhibiting caspases -3, -6, -7, -8, and -10). Treatment was initiated in 8-week old db/db mice and mice were analyzed after 12-weeks of treatment at the age of 20 weeks. Both compounds efficiently reduced the frequency of TUNEL positive glomerular cells (as previously shown refs 18 and 21), yet only minocycline, but not the caspase-inhibitor CIX, ameliorated markers of dNP (albuminuria and extracellular matrix accumulation, as reflected by the fractional mesangial area, FMA; Fig. 1a,c,d), implying that minocycline protects from dNP independent of apoptosis inhibition. Blood glucose levels, body weight, and liver enzymes did not differ among experimental mice (Fig. 1b, Supplementary Fig. S5a and data not shown). Minocycline likewise protected C57BL/6 mice with streptozotocin-induced hyperglycemia from developing hallmarks of dNP (Supplementary Fig. S1), corroborating previous data^18^. Again, minocycline had no effect on blood glucose levels, body weight, or liver enzymes (Supplementary Figs S1 and S5b). Taken together, minocycline provides nephroprotection in experimental dNP in both type 1 and type 2 diabetes models independent of apoptosis inhibition.

### Inflammasome inhibition by minocycline *in vivo*

**Figure 2:**
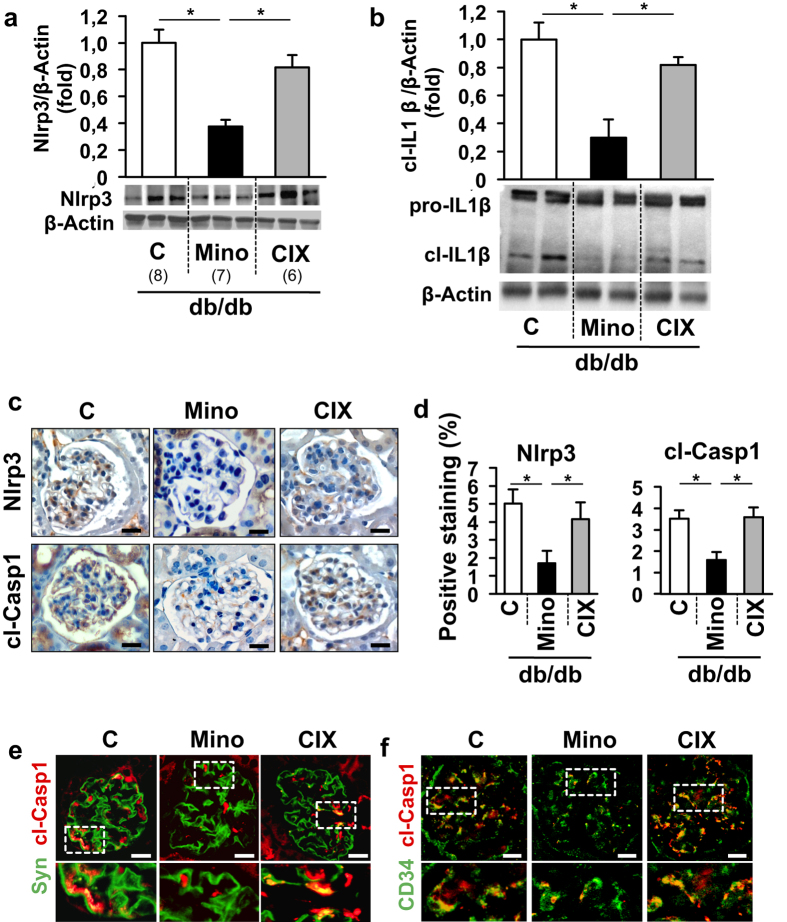
Minocycline prevents glomerular inflammasome activation. (**a,b**) In db/db mice treated with minocycline, but not in those treated with CIX, expression of Nlrp3 as well as the cleaved form of IL1β (cl-IL1β, **b**) are reduced in renal cortex extracts. (**c,d**) Immunohistochemical detection of Nlrp3 and cleaved caspase-1 demonstrates that minocycline, but not CIX, reduces markers of inflammasome activation within diseased glomeruli of db/db mice. (**e,f**) In glomeruli cleaved caspase-1 (cl-Casp1, red, **e,f**) co-localizes with the podocyte marker synaptopodin (Syn, green, **e**) and the endothelial marker CD34 (green, **f**). Minocycline, but not CIX application, reduces co-localization for both cell-types. Mean values ± SEM (**a,b,d**).; size bar (**c,e**): 20 μm; *P < 0.05 (**a,b,d**: ANOVA). Number of mice in each group is shown in parentheses in (**a**); (**a,b**) Representative cropped images without further modification are shown; exemplary uncropped images are provided in Supplementary Fig. 6.

Recently, sterile inflammation rather than cell-death has been linked with dNP^21^,^22^. Of note, the polycaspase-inhibitor CIX does not target caspase-1, the interleukin-1 converting enzyme that is required for inflammasome activation. This raises the question as to whether minocycline ameliorates dNP by targeting the inflammasome. To evaluate this question we determined markers of inflammasome activation in db/db mice. In renal cortex extracts of db/db mice minocycline, but not CIX, reduced Nlrp3 expression and IL1β cleavage (Fig. 2a,b). Immunohistochemical assessment revealed a marked reduction of glomerular Nlrp3 and cleaved caspase-1 in minocycline but not in CIX receiving mice compared to control db/db mice (Fig. 2c,d). We observed colocalization of cleaved caspase-1 with podocytes and glomerular endothelial cells (GEnC) in histological sections of db/db mice (Fig. 2e,f and Supplementary Fig. S2a). Minocycline, but not CIX, markedly reduced glomerular (podocyte and GEnC) specific inflammasome activation (Fig. 2e,f and Supplementary Fig. S2b). These data establish that minocycline dampens diabetes induced renal and glomerular inflammasome activation.

### Minocycline inhibits glucose-induced inflammasome activation in glomerular cells and normalizes glomerular filtration *in vitro*

**Figure 3:**
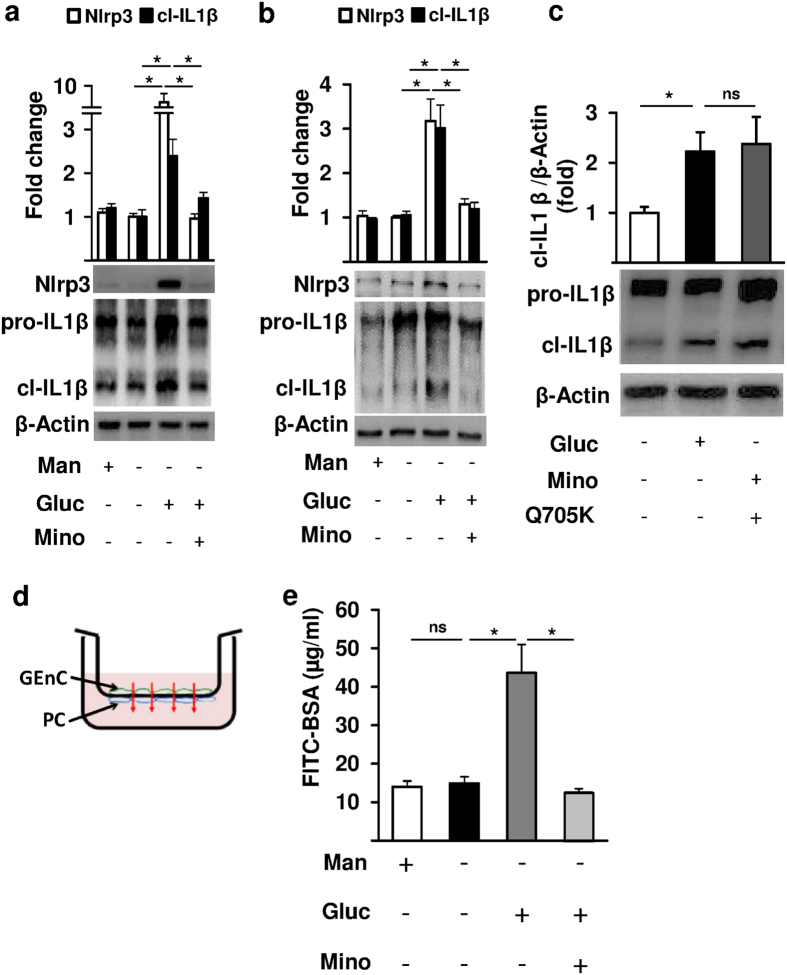
Minocycline prevents inflammasome activation in glucose challenged podocytes and glomerular endothelial cells (GEnC) *in vitro*. (**a,b**) High glucose (25 mM, 24 h), but not mannitol (25 mM, 24 h), induces the inflammasome markers Nlrp3 and cleaved IL1β in podocytes and glomerular endothelial cells *in vitro*. Minocycline (10 μM, 24 h) abrogates glucose-induced inflammasome activation. (**c**) Following transfection of human podocytes with a human Nlrp3 mutant (Q705K), resulting in constitutive active Nlrp3, minocycline (10 μM, 24 h) fails to suppress IL1β cleavage. (**d,e**) Minocycline prevents glucose-induced glomerular filtration barrier disruption *in vitro*; scheme reflecting the *in vitro* model used to mimic the glomerular filtration barrier (**e**) and bar graph summarizing results for albumin concentration in the lower chamber 3 hours after the addition of FITC-BSA to the upper chamber. Mean values ± SEM (**a–c,e**); *P < 0.05, ns: non-significant (**a–c**: Mann-Whitney-test; e: t-test); (**a–c**) representative cropped images without further modification are shown; exemplary uncropped images are provided in Supplementary Fig. 6.

To determine whether minocycline cell-autonomously inhibits inflammasome activation in podocytes and GEnC we incubated glucose challenged podocytes and GEnC with minocycline *in vitro*. Glucose induced Nlrp3 expression and caspase-1 activation in podocytes and GEnC, reflecting inflammasome activation^22^. Glucose-dependent inflammasome activation was prevented by minocycline both in podocytes and GEnCs (Fig. 3a,b). The effect of minocycline in regard to IL1β maturation was comparable to that of a caspase-1 inhibitor (Supplementary Fig. S3). Transfection with a constitutively active Nlrp3 mutant (Q705K) abolished minocycline’s suppressive effect on IL1β maturation in glucose stressed podocytes (Fig. 3c), demonstrating that minocycline mediated inhibition of IL1β-cleavage depends on Nlrp3.

To determine the effect of glucose-induced inflammasome activation and its inhibition by minocycline on the function of the glomerular filtration barrier we used a recently established *in vitro* model mimicking the glomerular filtration barrier (Fig. 3d and method section). Glucose, but not mannitol, markedly increased the transport of albumin across the *in vitro* filtration barrier consisting of podocytes and GEnC. The barrier disruptive effect of glucose was inhibited by minocycline (Fig. 3d,e), indicating that minocycline stabilizes the function of the glomerular filtration barrier through a direct effect on its cellular components.

### Minocycline inhibits oxidative stress

**Figure 4:**
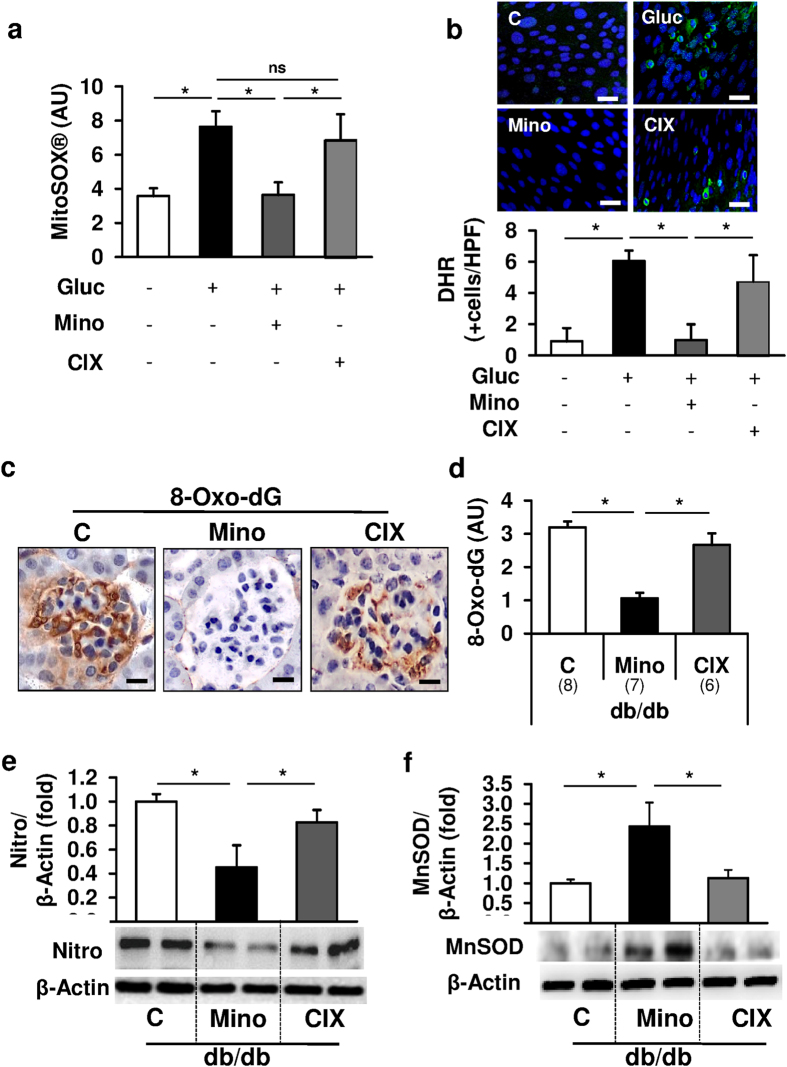
Minocycline prevents glucose-induced ROS formation. (**a,b**) Minocycline (10 μM, Mino, 24 h), but not CIX (5 μM, 24 h), prevents mitochondrial ROS formation (a: MitoSOX, b: Dihydrorhodamine, DHR, green) in glucose stressed murine podocytes *in vitro*. (**c–f**) Application of minocycline, but not CIX, prevents glomerular accumulation of the ROS marker 8-Oxo-2′-deoxyguanosine (8-Oxo-dG, **c**,**d**) and decreased nitrotyrosine (Nitro, **e**) levels while increasing mitochondrial manganese superoxide dismutase (MnSOD, **f**) in renal cortex extracts in 20 weeks old db/db mice as compared to PBS-receiving db/db controls (C). Representative 8-Oxo-2′-deoxyguanosine (8-Oxo-dG) stained images detected by HRP-DAB reaction, brown; hematoxylin counterstain, blue; size bar: 20 μm (**c**). Mean values ± SEM (**a–f**). Size bars (**b,c**): 20 μm; *P < 0.05 (**a,b,d–f**: ANOVA). Number of mice in each group is shown in parentheses in (**d–f**) representative cropped images without further modification are shown; exemplary uncropped images are provided in Supplementary Fig. 6.

Mitochondrial ROS can activate the inflammasome and minocycline has been shown to target mitochondria^16^,^25^. Hence we next evaluated whether minocycline inhibits diabetes-associated inflammasome activation by reducing mitochondrial ROS-generation. Indeed, in glucose stressed podocytes minocycline, but not the caspase-inhibitor CIX, prevented glucose-induced mitochondrial ROS generation, as reflected by staining with MitoSOX^®^ and dihydrorhodamine (DHR) (Fig. 4a,b), two dyes detecting mitochondrial ROS-production. Similarly, treatment with minocycline, but not with CIX, markedly reduced glomerular 8-Oxo-dG (8-Oxo-2′-deoxyguanosine) staining and nitrotyrosine levels (Fig. 4c–e) while increasing levels of the mitochondrial antioxidant manganese superoxide dismutase (MnSOD) in renal cortex extracts of db/db mice (Fig. 4f). Thus minocycline conveys an antioxidant effect in glucose stressed podocytes and diabetic kidneys. This suggests that minocycline restricts glucose-induced inflammasome activation by inhibiting ROS generation. However, the mechanism through which minocycline restricts ROS generation and thus inflammasome activation remains unknown.

### Stabilization of Nrf2 by minocycline

The transcription factor Nrf2, which initiates antioxidant defense mechanisms, ameliorates dNP in mice^10^,^11^. As increased ROS generation causes inflammasome activation and aggravates dNP we hypothesized that minocycline restricts glucose-induced inflammasome activation through a Nrf2-dependent mechanism^22^. First, to determine whether Nrf2 modulates glucose induced inflammasome activation we reduced Nrf2 expression *via* knock down in podocytes. Loss of endogenous Nrf2 expression markedly enhanced the glucose induced inflammasome activation, as reflected by the increase of cleaved IL1β, as compared to glucose challenged control (non-specific siRNA) podocytes (Supplementary Fig. S4), establishing that Nrf2 restricts glucose-induced inflammasome activation in podocytes.

**Figure 5:**
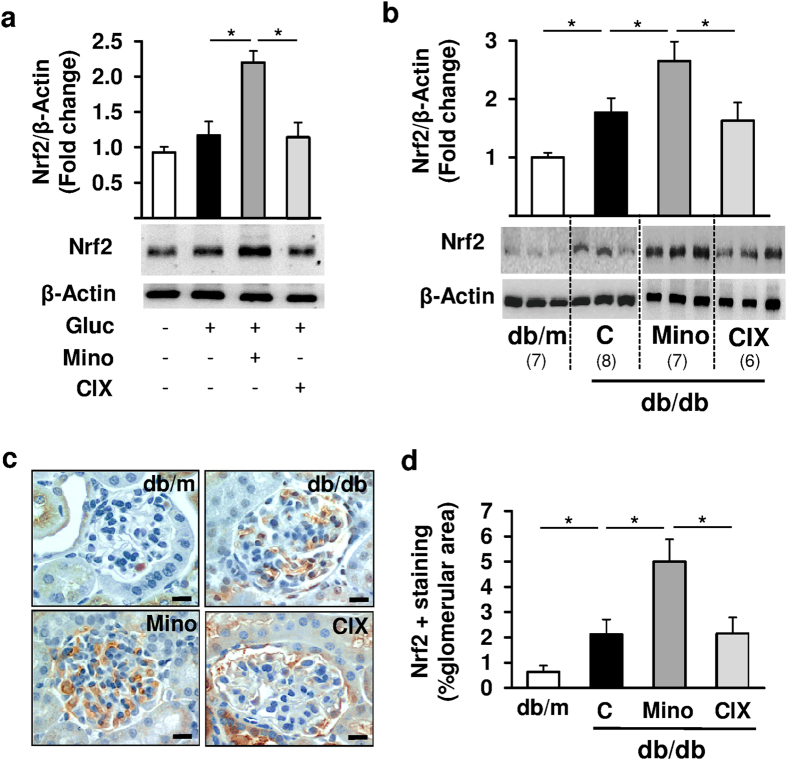
Minocycline increases Nrf2 expression in glucose challenged podocytes *in vitro* and in diabetic mice *in vivo*. (**a**) In glucose stressed murine podocytes minocycline (10 μM, 24 h), but not CIX (5 μM, 24 h), increases Nrf2 protein levels *in vitro*. (**b–d**) In 20 weeks old db/db mice minocycline, but not CIX, induces Nrf2 protein levels in renal cortex extracts and in glomeruli (**c,d**) as compared to db/db control (C) mice. Representative Nrf2 stained images detected by HRP-DAB reaction, brown; hematoxylin counterstain, blue; size bar: 20 μm (**c**). Mean values ± SEM (**a–d**). *P < 0.05 (**a**: Mann-Whitney-test; **b,d**: ANOVA). Number of mice in each group is shown in parentheses in (**b**); (**a,b**) representative cropped images without further modification are shown; exemplary uncropped images are provided in Supplementary Fig. 6.

To determine whether minocycline modulates Nrf2 expression we analyzed glucose stressed podocytes. Exposure to high glucose concentration slightly (although not significantly) induced Nrf2 expression, which was further induced by minocycline, but not by the caspase-inhibitor CIX (Fig. 5a). In line with these *in vitro* results minocycline, but not the caspase-inhibitor CIX, induced Nrf2 expression in the renal cortex (Fig. 5b) and in glomeruli (Fig. 5c,d) of db/db compared to untreated db/db and db/m control mice. These data demonstrate that minocycline increases endogenous Nrf2 expression in dNP.

**Figure 6:**
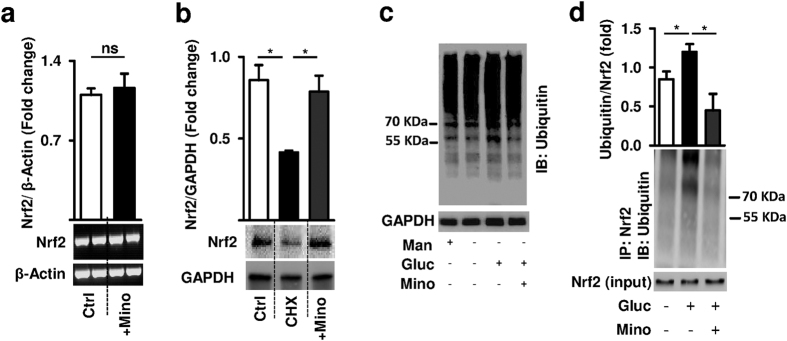
Minocycline increases Nrf2 protein stability and suppresses Nrf2 ubiquitination. (**a**) Minocycline (+Mino) has no impact on Nrf2 mRNA expression in murine podocytes compared to untreated controls (Ctrl). (**b**) After incubation of podocytes with the protein synthesis inhibitor cycloheximide (CHX, 10 mg/ml for 24 h) Nrf2 protein levels decline. Application of minocycline stabilizes Nrf2 levels. (**c**) After glucose challenge (25 mM for 24 h) protein ubiquitination (IB: Ubiquitin) is increased compared to unstressed or mannitol incubated (25 mM for 24 h) controls. Minocycline reduces glucose-induced protein ubiquitination; representative immunoblot. (**d**) Minocycline reduces glucose-induced Nrf2 ubiquitination. Immunoprecipitation (IP) of Nrf2 from whole cell lysates of control (5 mM glucose), glucose challenged (25 mM for 24 h), or glucose plus minocycline (10 μM, 24 h) exposed podocytes. Mean values ± SEM (**a,b,d**); *P < 0.05, ns: non-significant (**a,b,d**: Mann-Whitney-test). (**a–d**) Representative cropped images without further modification are shown; exemplary uncropped images are provided in Supplementary Fig. 6.

The above immunoblotting and immunohistochemical analyses demonstrate increased Nrf2 protein levels following minocycline treatment *in vitro* and *in vivo*. To further elucidate the underlying mechanism we determined Nrf2 mRNA expression in minocycline treated podocytes (Fig. 6a). Minocycline did not affect Nrf2 mRNA expression (Fig. 6a), indicating that Nrf2 is regulated posttranslationally by minocycline. Posttranslational regulation of Nrf2 stability *via* ubiquitination and protein degradation is well established^26^,^27^. To determine Nrf2 protein stability we blocked its resynthesis using cycloheximide (CHX, Fig. 6b). In the presence of cycloheximide Nrf2 protein levels declined, but Nrf2 protein levels remained stable if minocycline was added in addition to cycloheximide (Fig. 6b). This suggests that minocycline regulates Nrf2 *via* the ubiquitin-proteasomal system. Indeed, minocycline reduced overall glucose-induced ubiquitination (Fig. 6c) and–importantly–it specifically prevented glucose induced Nrf2 ubiquitination in podocytes (Fig. 6d). These results suggest that minocycline maintains Nrf2 protein levels in glucose stressed podocytes by limiting ubiquitination and proteasomal degradation of Nrf2.

### Nrf2 is required for minocycline dependent nephroprotection

**Figure 7:**
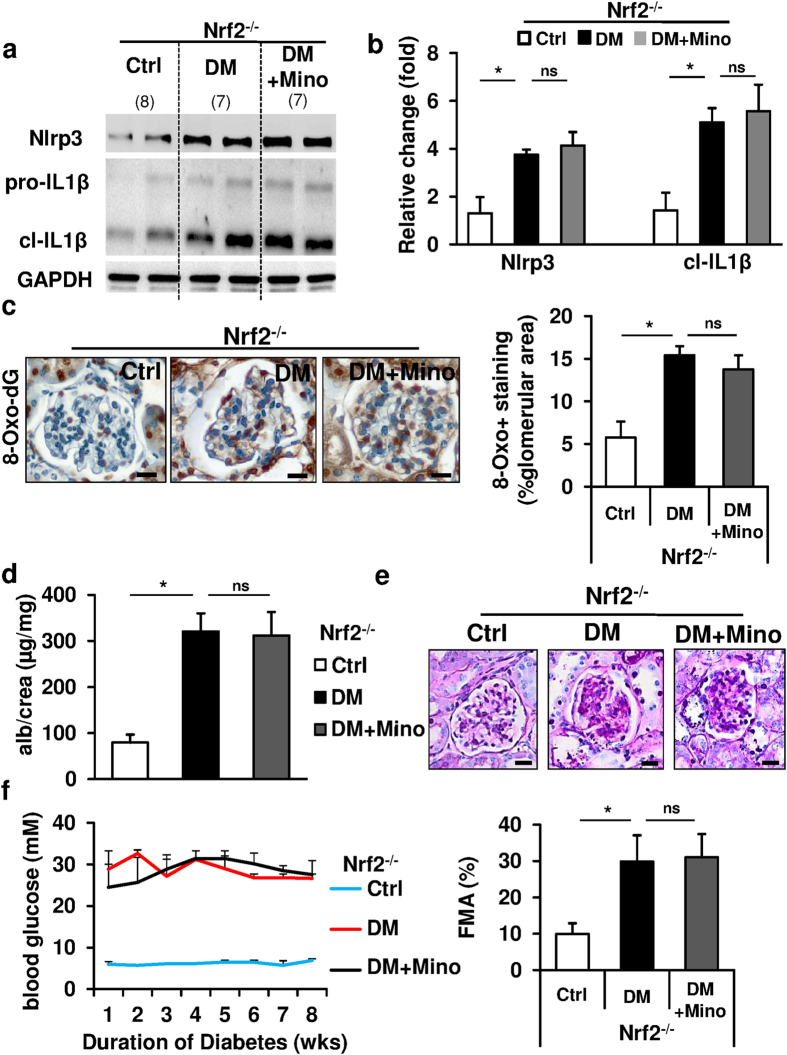
Nrf2 is required for minocycline-mediated protection from diabetic nephropathy. (**a–c**) Nrf2 deficiency (Nrf2^−/−^) abolishes minocycline’s inhibitory effect on Nlrp3 (**a**), cl-IL1β (**b**) and glomerular 8-Oxo-2′-deoxyguanosine (8-Oxo-dG) (**c**) induction in diabetic C57BL/6 mice (DM). (**d,e**) Minocycline fails to reduce albuminuria (**d**) and fractional mesangial area (FMA, representative PAS-stained histological images, **e**) in Nrf2^−/−^ mice. (**f**) Glucose levels are comparable in diabetic Nrf2^−/−^ mice without (DM) or with (DM + Mino) minocycline application. Representative immunoblots (**a**) and bar graphs summarizing results (**b–d,f**). Representative 8-Oxo-2′-deoxyguanosine (8-Oxo-dG) stained images detected by HRP-DAB reaction, brown; hematoxylin counterstain, blue; size bar: 20 μm (**c,e**). Ctrl indicates non-diabetic Nrf2^−/−^ control mice. Mean value ± SEM (**b–f**); *P < 0.05, ns: non-significant (**b–e**: t-test). Number of mice in each group is shown in parentheses in (**a**); (**a**) representative cropped image without further modification is shown; exemplary uncropped images are provided in Supplementary Fig. 6.

To ascertain that the nephro- and cytoprotective effect of minocycline depends on Nrf2 *in vivo* we determined the effect of minocycline on dNP, inflammasome activation, ROS-generation, and glomerular cell death in Nrf2 deficient (Nrf2^−/−^) diabetic mice. In Nrf2^−/−^ diabetic mice minocycline failed to reduce Nlrp3 expression, IL1β cleavage or glomerular 8-Oxo-2′-deoxyguanosine (8-Oxo-dG) (Fig. 7a–c). Furthermore, minocycline failed to protect Nrf2^−/−^ mice from dNP, as indicated by a failure to reduce albuminuria and extracellular matrix accumulation (Fig. 7d,e). Again, minocycline had no impact on blood glucose levels in Nrf2^−/−^ diabetic mice (Fig. 7f). Taken together, the cyto- and nephroprotective effect of minocycline depends on Nrf2, which is stabilized by minocycline posttranslationally and reduces ROS-generation and inflammasome activation in renal cells.

## Discussion

Within the current study we provide new insights into the nephroprotective mechanism of minocycline in dNP. Minocycline reduces glomerular ROS-generation and inflammasome activation *via* the transcription factor Nrf2. Functionally, minocycline protects the endothelial-podocyte filtrations barrier *in vitro* and ameliorates diabetic kidney disease *in vivo*. Mechanistically minocycline maintains renal protein levels of Nrf2, possibly by reducing its ubiquitination and degradation. The inhibition of inflammasome activation *via* Nrf2 identifies a new minocycline-dependent cytoprotective mechanism that is of potential translational relevance in dNP, but may also have implications beyond dNP^28^,^29^. Furthermore, the protection from dNP through inflammasome inhibition and the failure of the poly-caspase inhibitor CIX (targeting caspases-3,-6,-7,-8,-10, but not caspase-1) strengthens the recently described pathogenetic role of inflammasome activation and sterile inflammation in dNP while supporting the notion that caspase-3-dependent apoptosis is negligible^21^,^22^.

Sterile inflammation is an established hallmark of dNP and is increasingly recognized as a disease aggravating mechanism in dNP. Inflammasome activation has been mechanistically linked with excess ROS generation^30^. Hence, a crucial pathophysiological role of inflammasome mediated sterile inflammation is entirely compatible with excess ROS generation, which is considered to be a unifying pathomechanism in dNP^31^,^32^. However, while inhibition of excess ROS-generation is beneficial in experimental dNP, clinical studies evaluating antioxidants failed to demonstrate a benefit^33^. The failure of clinical studies using non-selective ROS scavengers has been attributed to the inadvertent inhibition of physiological ROS functions^34^. Hence, alternative approaches aiming to restrict excess ROS formation are needed. Possible approaches include the inhibition of specific ROS sources, e.g. mitochondrial ROS^35^, or the amplification of endogenous ROS-scavenging pathways, e.g. by inducing the redox-sensitive transcription factor Nrf2^10^. Alternatively, targeting ROS-dependent pathomechanisms, such as inflammasome activation, may allow nephroprotection without interfering with the physiological functions of ROS. Inflammasome inhibition provides nephroprotection in several renal injury models and amelioration of dNP in human diabetic patients has been reported in few cases^21^,^22^,^36^,^37^.

Based on the current results minocycline may be an interesting therapeutic approach to restrict ROS-mediated inflammasome activation in dNP. We establish that minocycline reduces ubiquitination of Nrf2 and increases Nrf2 protein stability. Nrf2 co-ordinately induces the expression of antioxidant defence-enzymes, such as NQO1, NQO2, HO-1, and MnSOD^8^,^38^. The regulation of Nrf2 in murine models of dNP, as observed in the current studies, raises the question as to whether minocycline may simultaneously target other pathways involved in dNP. Thus, Nrf2 interacts with Notch1 and NF-kB signaling and hence with pathways thought to be relevant in dNP^39^,^40^. Intriguingly, just like Nrf2 both Notch1 and NF-kB are regulated by ubiquitination. Further studies are required to evaluate whether minocycline targets multiple pathways linked with dNP, potentially *via* ubiquitination^2^,^3^,^4^. The versatility of minocycline allowing it to target multiple pro-inflammatory pathways may be an important factor for its well-established cyto-protective effects. Furthermore, recent evidence suggests that multiple cellular stress pathways, such as mitochondrial, inflammatory and oxidative stress, need to be simultaneously therapeutically targeted to yield a favourable outcome in dNP^41^. Therefore targeting Nrf2 and interacting pathways with a well-tolerated drug such as minocycline could prove to be a promising approach to dNP.

Pre-clinical studies using Nrf2 activators or Nrf2 deficient mice and *in vitro* work established a crucial function of Nrf2 in modulating glucose induced ROS generation and suggest that Nrf2 activators may be a suitable pharmaceutical approach to limit glucose-induced excess ROS generation^42^,^43^,^44^,^45^ and to convey nephroprotection in diabetes^10^,^11^. However, after initial promising results in clinical studies^12^,^46^ a phase III study evaluating the Nrf2 activator bardoxolone in type 2 diabetic patients with stage IV CKD (chronic kidney disease) was prematurely stopped because of increased of heart failure rates in the treatment group, which may reflect off-target effects^6^,^13^. Minocycline may present an interesting alternative in this regard. Minocycline has been in clinical use for decades, e.g. as a long-term treatment for acne in adolescence or for gingivitis in diabetic patients, and is generally well-tolerated^47^,^48^,^49^. Thus, clinical experience and safety data on minocycline are on hand. Correspondingly, minocycline was well tolerated in the current rodent study, as mice did not show any obvious signs of toxicity and liver enzymes (ALT, AST) remained stable (Supplementary Fig. S5). This contrasts a previous study using the bardoxolone methyl analogue RTA 405 in ZDF rats, in which liver toxicity and an impaired physical status of RTA 405 treated animals was observed^50^. In addition, minocycline is inexpensive and readily available and, based on the current results, targets crucial pathomechanisms in dNP (excess ROS formation and sterile inflammation). Interestingly, small pilot studies demonstrated that oral application of minocycline is not only a safe, but also an effective treatment in diabetic patients with macular edema and neuropathy^28^,^29^. Very recently, the anti-proteinuric effect of minocycline was prospectively evaluated in 14 patients with dNP^17^. After 24 weeks of minocycline treatment a non-significant reduction of urine protein/creatinine and microalbumin/creatinine ratios was apparent in the minocycline, but not in the placebo treated group^17^. Interestingly, inflammatory markers (urine IL6 and osteoprotegerin) were significantly reduced in the minocycline group, which is in agreement with the anti-inflammatory effect of minocycline identified within this study. Based on the current and this recent clinical pilot study sufficiently powered studies, possibly for longer period, evaluating the efficacy of minocycline in patients with dNP are now warranted^17^,^18^,^19^.

The mouse models used in this study for diabetic nephropathy are known to develop functional and morphological renal abnormalities^51^,^52^. However, in comparison to the human disease only features of early diabetic nephropathy are replicated. The typical nodular glomerulosclerosis of late stage dNP in humans is not apparent in STZ induced diabetic C57BL/6 or db/db mice^51^. Therefore the potential of minocycline to target advanced pathological features of human dNP cannot be assessed. Future studies using mouse models of more advanced dNP, such as eNOS-deficient mice^53^,^54^, may be better suitable to evaluate the therapeutic benefit of minocycline in advanced dNP. Alternatively, observational clinical studies may provide insights into the role of minocycline in advanced dNP, e.g. in patients with established dNP and receiving minocycline for a different indication (e.g. acne or infections). Furthermore, the question whether minocycline may provide an added value on top of established therapies such as ACE-inhibition or reverses established dNP remains to be addressed in future studies.

The cytoprotective effects of minocycline, which was originally identified as a tetracycline-derived antibiotic, are well documented and have been linked with modulation of mitochondrial function, normalization of the Bax/Bcl-2 ratio, reduced cytochrome C release, and apoptosis inhibition^14^,^15^. However, the exact mechanism of minocycline mediated cytoprotection remains unknown. By linking minocycline’s protective effect with sustained Nrf2 protein levels and reduced Nrf2 ubiquitination the current data provides new insights into the cytoprotective action of minocycline. The importance of the ubiquitin-proteasomal system for the regulation of Nrf2 is well established^7^ and hence it appears plausible that the minocycline mediated reduction of Nrf2 ubiquitination is functionally relevant. We acknowledge that the exact mechanism through which minocycline inhibits Nrf2 ubiquitination and the relevance of this for Nrf2 protein stabilization or sub-cellular localization remain to be evaluated^55^. Of note, the current results are in line with recent reports demonstrating that proteasomal inhibitors induce anti-oxidant effects^56^. Furthermore, the proteasomal inhibitor MG132 conveys nephroprotective effects in diabetic rats, which have been linked with increased Nrf2, superoxide dismutase-1 (SOD1), catalase (CAT), and glutathione peroxidase (GPx) expression and reduced nitrotyrosine levels in the kidney of diabetic rat^57^. However, while in the latter study the causality between increased Nrf2 expression and nephroprotection by MG132 remained unanswered, we demonstrate a mechanistic relevance of sustained Nrf2 expression for minocycline mediated nephroprotection using genetically Nrf2 deficient mice. Based on the current results minocycline posttranslationally upregulates Nrf2 in glucose stressed podocytes *in vitro* by inhibiting its glucose-induced ubiquitination. Further studies are needed to identify the mechanism through which minocycline limits glucose induced Nrf2 ubiquitination, e.g. the potential involvement of deubiquitinating enzymes. Such mechanistic details will then allow further studies, e.g. podocyte specific inhibition of the corresponding deubiquitinating enzyme, to provide *in vivo* evidence for the proposed mechanism.

The association of dNP and sterile inflammation is now established^21^,^22^. Based on the current and previous work we propose that glucose induced ROS and inflammasome activation drive the disease processes in dNP^21^,^22^,^57^. Therapeutic approaches directly targeting the inflammasome or controlling the inflammasome indirectly by restricting unchecked ROS generation *via* Nrf2 and/or limiting proteasomal degradation of Nrf2, as shown here for minocycline, may constitute promising new approaches to combat onset and progression of dNP.

## Material and Methods

### Mice

Nrf2^−/−^ (Nfe2l2), db/db (Lepr db/db), and non-diabetic db/m control mice (C57BL/6J background) were obtained from Jackson Laboratories, Bar Harbor, ME, USA. In the current study we used littermates which have been backcrossed for at least 10 generations on a C57BL/6J background. All animal experiments were conducted in accordance with the relevant guidelines by the local Animal Care and Use Committee and approved by the local Animal Care and Use Committee (Landesverwaltungsamt Halle, Germany; Licences: 2-1085, 2-1179 and 2-1104).

### Diabetic nephropathy models

We used two different mouse models of dNP within the current study. First, we use the db/db mice, in which treatment was initiated at age 8-weeks and mice were sacrificed after 12 weeks of treatment at the age of 20 weeks. In non-treated db/db mice indices of dNP, e.g. albuminuria and glomerular extracellular matrix accumulation (FMA, fractional mesangial area), are markedly increased in 20 weeks old db/db mice^58^.

In addition we used the streptozotocin (STZ) plus unilateral nephrectomy model of dNP^22^,^59^,^60^. In this model diabetes was induced by injections of STZ (i.p., 40 mg/kg body weight, freshly dissolved in 0.05 M sterile sodium citrate, pH 4.5) for five consecutive days two weeks after unilateral nephrectomy. Age-matched control mice received 100 μL PBS i.p. for five consecutive days. Mice were considered diabetic if blood glucose levels were above 300 mg/dL 16 d after the last STZ injection. Blood and tissue samples were obtained after 10 weeks of persistent hyperglycemia in uni-nephrectomised diabetic mice. Age-matched littermates served as controls.

### *In vivo* intervention studies

A subset of mice was treated with the poly-caspase inhibitor CIX (peptide sequence: Ac-DEVD-CMK) (20 mg/kg body weight daily; intraperitoneally), known not to affect IL1β processing (Merck, Millipore) or minocycline (5 mg/kg body weight daily, intraperitoneally)^18^,^61^. Control mice received an equal volume of PBS. Interventions with minocycline or CIX or were initiated in 8 weeks old db/db mice to determine its preventive effect. In uninephrectomized, STZ treated mice treatment with minocycline was initiated 2 weeks after establishment of stable hyperglycemia and continued for 10 weeks. Control mice were injected with PBS.

### Histology and immuohistochemical analysis

Histology and immunohistochemical analysis was performed as previously described^18^,^22^,^59^,^62^. Freshly sacrificed mice were first perfused with ice cold PBS (10 ml) and then with 4% buffered paraformaldehyde (5 ml). Tissues were furthered fixed with 4% buffered paraformaldehyde for 2 days at 4 °C, then embedded in paraffin and processed for sectioning. Extracellular matrix deposition in glomeruli was assessed by Periodic acid–Schiff staining. Histological evaluations were performed in accordance with the Diabetes Complications Consortium guidelines (DCC) (www.diacomp.org). The fractional mesangial area (FMA) was calculated following the current DCC protocol. Briefly, 4–5 μm thick sections were stained with Periodic acid-Schiff reagent. At least 30 different superficial glomeruli/mouse were randomly chosen for analysis. For every investigated glomerulus, total glomerular area and glomerular tuft area were determined by tracing the outline of the Bowman’s capsule and the tuft, respectively, using ImageJ. The FMA was calculated as the percentage of the glomerular area relative to the tuft area^22^,^59^. Immunohistochemical detection of anti-8-oxoguanine, Nrf2, Nlrp3, synaptopodin, cleaved caspase 1, or nitrotyrosine was performed using corresponding specific primary and secondary antibodies. Control images, obtained following incubation with non-specific primary antibodies, were used for background correction. Specificity of caspase-1 and Nlrp3 antibodies was confirmed using sections of corresponding knock-out mice as negative controls. The Image Pro Plus software (version 6.0) and Image J software were used for image analysis. All histological analyses were performed by two independent blinded investigators. Immunohistochemistry and immunofluorescence images were captured with an Olympus Bx43-Microscope (Olympus, Hamburg, Gemany). Confocal images were obtained at 40x magnification using a Zeiss LSM 4 Pascal microscope (Carl Zeiss, Jena, Germany).

### Determination of albuminuria and liver enzymes

Mouse urine albumin and creatinine were measured as previously described^22^,^59^. Briefly, the day before sacrificing animal at the end of experimental time, mice were placed individually in metabolic cages and 12 hour urine samples were collected. Urine albumin was determined using an ELISA for mouse albumin according to the manufacturer’s instructions, and urine creatinine was determined using a commercially available assay of a modified version of the Jaffe method (X-Pand automated platform, Siemens). Serum activity of the liver enzymes ALT (alanin aminotransferase) or AST (aspartat aminotransferase) was determined according to current protocols by the IFCC (International Federation of Clinical Chemistry) with a pyridoxalphosphate-dependent UV-reaction at 37 °C.

### Cell culture, Nrf2 knockdown and Nlrp3 gain of function

Conditionally immortalized human and mouse wild-type podocytes were cultured as described previously^18^,^62^. In brief, podocytes were grown on 10 cm^2^ cell culture plates coated with 0.2% collagen type 1 at 33 °C in the presence of interferon-γ (10 U/ml) to enhance expression of the thermosensitive T antigen. Under these conditions, cells proliferate and remain undifferentiated. To induce differentiation, podocytes were grown at 37 °C in the absence of interferon-γ for 14 days. Experiments were performed after 14 days of differentiation. Differentiation was confirmed by determining expression of synaptopodin and Wilms tumor-1 protein. 293T cells (American Type Culture Collection, Rockville, MD) were cultured in DMEM with 10% FBS and penicillin/streptomycin. Mouse glomerular endothelial cells (GEnC) were obtained from J. Anders (Munich, Germany). GEnC were maintained in RPMI medium with 10% FCS and 1% antibiotics at 37 °C in a humidified 5% CO2 incubator.

Mouse Nrf2 gene expression was knocked down using siRNA against Nrf2 gene transcripts. Briefly, 0.25–1 × 10^6^ cells were seeded in 6 well culture plate. Upon reaching 60–80% confluence, siRNA (final conc. 30p mol) was added to diluted lipofectamine^®^RNAiMAX reagent in 1:1 ratio. Cells were then incubated for 5 minutes at room temperature. siRNA lipid complex (final conc. 25 pmol) was added to cells and Incubated for 3 days at 37 °C. The transfection efficiency was ascertained by Nrf2 protein expression, as determined by immunoblotting.

A Nlrp3 mutant resulting in expression of an constitutive active IL1β was used to generate cells with a gain-of-function of Nrlp3, as previously described^22^.

### Glomerular filtration barrier *in vitro* assay

To determine glomerular filtration barrier we used a recently established *in vitro* model mimicking central aspects of the glomerular filtration barrier with some modifications^63^,^64^. This model consists of a polyethyleneterephthalate (PET) membrane (Millipore membrane, Millicell Hanging Cell Culture Insert) with 1 μm pores that is coated on both sides with collagen type IV. First inserts were placed upside-down in a larger well and podocytes were seeded on the membrane-side facing upwards. Once attached (~6 h), the insert was turned around, placed in a well of appropriate size, and podocytes were allowed to differentiate for 10 days. Then GEnC were placed on the side of the membrane now facing upwards. Cells were cultured under resting conditions for additional four days using podocyte and endothelial cell specific medium in the corresponding chamber. This *in vitro* model enables functional permeability assays by determining the transmembrane passage of fluorescently labeled proteins with a defined molecular weight (such as FITC-labelled albumin). To mimic hyperglycemic conditions corresponding medium containing 25 mM of glucose was added to both chambers, while control cells were kept at low glucose concentrations (5 mM). After 48 hours medium in both chambers was changed to serum-free medium (SFM, RPMI 1640). After 1 h FITC-labeled BSA (Sigma) was added to the upper chamber at a final concentration of 0.5 mg/ml. After further 3 h the concentration of FITC-labeled BSA in the lower chamber was measured. The fluorescence was measured using fluorometric spectrophotometer at 495 nm and the concentration of FITC-BSA was calculated by reference to a set of standard dilutions. The amount of FITC-labeled BSA accumulating in the lower chamber reflects the barrier permeability.

### Immunoblotting and Immunoprecipitation

Immunoblotting and Immunoprecipitation were performed as previously described^18^,^22^,^59^,^62^. In brief, for immunoblotting whole cell lysates were prepared in RIPA buffer (50 mM Tris at pH 7.4, 1% Nonidet P-40, 0.25% sodium deoxycholate, 150 mM NaCl, 1 mM EDTA, and 1 mM Na_3_VO_4_, supplemented with protease inhibitor cocktail). Lysates were centrifuged (10,000 × g for 20 min at 4 °C) and supernatant was kept, while the pellet containing debris was discarded. The protein concentration in supernatants was quantified using BCA reagent. Equal amounts of protein were electrophoretically separated on 10% (vol/vol) or 12.5% (vol/vol) SDS polyacrylamide gels, transferred to PVDF membranes, and probed with the desired primary antibodies overnight at 4 °C. Membranes were then washed with PBST and incubated with anti-mouse (1:2,000), anti-goat IgG (1:2000), anti-rat IgG (1:2000) or anti-rabbit IgG (1:2000) horseradish peroxidase-conjugated antibodies, as indicated. Blots were developed with the immobilon western chemiluminiscent HRP substrate. To compare and quantify levels of proteins, the density of each band was measured by using ImageJ software. Equal protein loading was confirmed by immunoblotting with β-actin or GAPDH antibody.

Immunoprecipitation of podocyte total cellular proteins was done as described elsewhere^62^. Total cellular proteins were extracted with RIPA containing complete protease inhibitor cocktail. Samples were sonicated (set at 10% of maximum power) for 10 sec followed by 1 min incubation on ice. This process was repeated five times. Lysates were combined with 5 μg of specific antibody and incubated overnight at 4 °C on rotating shaker. Immunoprecipitates were purified with protein A/G agarose beads and washed with PBS containing protease inhibitor cocktail. Immunoprecipitates were fractionated by SDS-PAGE (10%), transferred to membranes, and subjected to immunoblotting with appropriate primary and secondary antibodies as described above.

### Mitochondrial ROS production

Mitochondrial specific ROS were measured as described elsewhere^22^ using MitoSOX™ following manufacturer’s instructions. Briefly, mouse podocyte were grown in 96-wells cell culture plate. Upon reaching 90% confluence, cells were washed once with PBS and then incubated with 5 μM MitoSOX™ for 10 min at 37 °C in the dark. Cells were washed 3x with wash buffer (Hank’s balanced salt solution, HBSS) and mitochondrial specific ROS were measured using a fluorescent plate reader (excitation/emission of 510/580 nm).

### RT-PCR

RNA was isolated using Trizol reagent according to manufacturer’s instructions. cDNA was generated using 1 μg total RNA following treatment with DNAse (5U/5 μg RNA) followed by reverse transcription using RevertAid™ H Minus First Strand cDNA Synthesis kit (Fermentas, Heidelberg, Germany). PCR primers used for expression of Nrf2 analyses are as follows: forward 5′-CGCTGGAAAAAGAAGTGGGC-3′, reverse 5′-AGTGACTGACTGATGGCAGC-3′. PCR conditions were optimized (95 °C for 2 min, then 35 cycles of 94 °C, for 20 sec; 60 °C for 20 sec; 72 °C for 30 sec; final extension at 72 °C for 12 min) to detect the logarithmic increase of the amplimer, which was separated on a 1.8% agarose gel and visualized by ethidium bromide staining. Expression was normalized to β-actin. Reactions lacking reverse transcriptase served as negative controls.

### Statistical Analysis

The data are summarized as means ± SEM (standard error of the mean). Statistical analyses were performed with Student’s t-test, ANOVA, Mann-Whitney-Test, as appropriate, and post-hoc comparison with the method of Tukey. The Kolmogorov-Smirnov (KS) test or D’Agostino-Pearson-Normality-test were used to determine whether the data are consistent with a Gaussian distribution. StatistiXL (www.statistixl.com) and Prism 5 (www.graphpad.com) software were used for statistical analyses. All data presented involving cell culture is representative of at least three independent repeat experiments. Statistical significance was accepted at values of p < 0.05.

## Additional Information

**How to cite this article**: Shahzad, K. *et al*. Stabilization of endogenous Nrf2 by minocycline protects against Nlrp3-inflammasome induced diabetic nephropathy. *Sci. Rep*. **6**, 34228; doi: 10.1038/srep34228 (2016).

## Supplementary Material

Supplementary Information
