# Supplementary material for: Stabilization of endogenous Nrf2 by minocycline protects against Nlrp3-inflammasome induced diabetic nephropathy
Source: Sci Rep. 2016 Oct 10;6:34228. doi: 10.1038/srep34228 (PMC5056367; doi:10.1038/srep34228)

SUPPLEMENTARY LEGENDS AND FIGURES

Stabilization of endogenous Nrf2 by minocycline protects against Nlrp3-inflammasome induced diabetic nephropathy

Khurrum Shahzad[1,2]*, Fabian Bock[1,3]*, Moh'd Mohanad Al-Dabet[1]*, Ihsan Gadi[1], Sumra Nazir[1], Hongjie Wang[1,4], Shrey Kohli[1], Satish Ranjan[1], Peter R. Mertens[5], Peter P. Nawroth[6], Berend Isermann[1]

Running headline: Minocycline stabilizes Nrf2 in dNP

[1] Institute of Clinical Chemistry and Pathobiochemistry, Otto-von-Guericke-University, 39120 Magdeburg, Germany

[2] University of Health Sciences, Khayaban-e-Jamia Punjab, 54600**,** Lahore, Pakistan

[3] Department of Medicine, Vanderbilt University Medical Center, 37232 Nashville, Tennessee, United States

[4] Department of Cardiology, Tongji Hospital, Tongji Medical College, Huazhong University of Science and Technology, Wuhan, China

[5] Clinic of Nephrology and Hypertension, Diabetes and Endocrinology, Otto-von-Guericke University Magdeburg, 39120 Magdeburg, Germany

[6] Department of Internal Medicine I and Clinical Chemistry, German Diabetes Center (DZD), University of Heidelberg, 69120 Heidelberg, Germany

* Equal contributing first authors

Supplementary FIGURE LEGENDS

Supplementary Figure 1. Minocycline ameliorates dNP in C57BL/6 mice. Hyperglycemia was induced in 8 weeks old C57BL/6 mice and a subgroup of diabetic mice received minocycline (5mg/kg, DM + Mino) and compared to PBS-injected diabetic control mice (DM). Minocycline prevents the diabetes-induced increase of albuminuria (a) and the fractional mesangial area expansion (FMA) (b). Minocycline has no effect on blood glucose (BG, c) or body weight (BW, d) in diabetic animals. Representative PAS-stained histological images shown in b. Size bar (b): 20µm. Ctrl indicates non-diabetic wild type control mice. Mean values ± SEM (a-d). *P<0.05, ns: non-significant; (**a-d**: t-test). Number of mice in each group is shown in parentheses in **a.**

Supplementary Figure 2. Immunofluorescence images of glomeruli in minocycline and CIX receiving db/db mice. Single channel images (corresponding to Fig 2e) of minocycline (Mino) and poly-caspase-inhibitor (CIX) treated db/db mice showing cleaved-caspase-1 (cl-Casp1, red, a,b) and the podocyte marker synaptopodin (Syn, green, a) or the endothelial marker CD34 (green, b). Representative conventional immunofluorescence microscopy images. Size bar: 20 µm.

Supplementary Figure 3. Minocycline reduces IL1β maturation as efficient as a caspase-1 inhibitor. Glucose challenge (25mM, 24h), but not mannitol (25mM, 24h), increases the cleavage of IL1β (cl-IL1β) in murine podocytes *in vitro.* Minocycline (10µM, 24h) and a caspase-1-inhibitor (Casp-1-Inh, Z-WEHD-FMK, 5µM, 24h) inhibit IL1β-cleavage (cl-IL1β) in murine podocytes *in vitro* to a similar extend. Representative immunoblot of at least three independent repeat experiments with a bar graph summarizing results. Mean values ± SEM; *P<0.05, ns: non-significant; Mann-Whitney-test.

Supplementary Figure 4. Nrf2 deficiency increases IL1β cleavage in glucose stressed podocytes *in vitro*. Knock-down of Nrf2 (Nrf2-KD) increases glucose-induced (25mM, 24h) cleavage of IL1β (cl-IL1β, a,b). Efficient knock-down of Nrf2 shown in c. Representative immunoblots (a,c) of at least three independent repeat experiments and a bar graph summarizing results (b). C indicates wild-type high-

glucose stimulated cells. Ctrl indicates untreated control cells. Mean values ± SEM; *P<0.05 (**b**: Mann-Whitney-test).

Supplementary Figure 5. Liver enzymes in minocycline treated db/db mice. Treatment with minocycline (5 mg/kg body weight daily, intraperitoneally) and CIX (20 mg/kg body weight daily; intraperitoneally) does not increase ALT (alanin aminotransferase) or AST (aspartat aminotransferase) serum activity in db/db mice or STZ-treated C57BL/6 mice. Liver enzyme activity in U/l, C: diabetic controls, not treated with minocycline. Mean values ± SEM of seven mice in each group; *P<0.05 (t-test).

Supplementary Figure 6. Full images of cropped immunoblotting gels.
(a) Uncropped immunoblot image corresponding to Figure 2a; protein levels of Nlrp3.
(b) Uncropped immunoblot image corresponding to Figure 3a; protein levels of Nlrp3 and IL-1β.
(c) Uncropped immunoblot image corresponding to Figure 3b; protein levels of Nlrp3.
(d) Uncropped immunoblot image corresponding to Figure 3c; protein levels of IL-1β.
(e) Uncropped immunoblot image corresponding to Figure 4e; protein levels of Nitrotyrosine.
(f) Uncropped immunoblot image corresponding to Figure 4f; protein levels of MnSOD.
(g) Uncropped immunoblot image corresponding to Figure 5a; protein levels of Nrf2.
(h) Uncropped immunoblot image corresponding to Figure 5b; protein levels of Nrf2.
(i) Uncropped immunoblot image corresponding to Figure 6a; mRNA levels of Nrf2.
(j) Uncropped immunoblot image corresponding to Figure 7a; protein levels of Nlrp3 and IL-1β.
Some gels were cut to save protein lysates prior to the protein transfer.

Supp. Fig 1

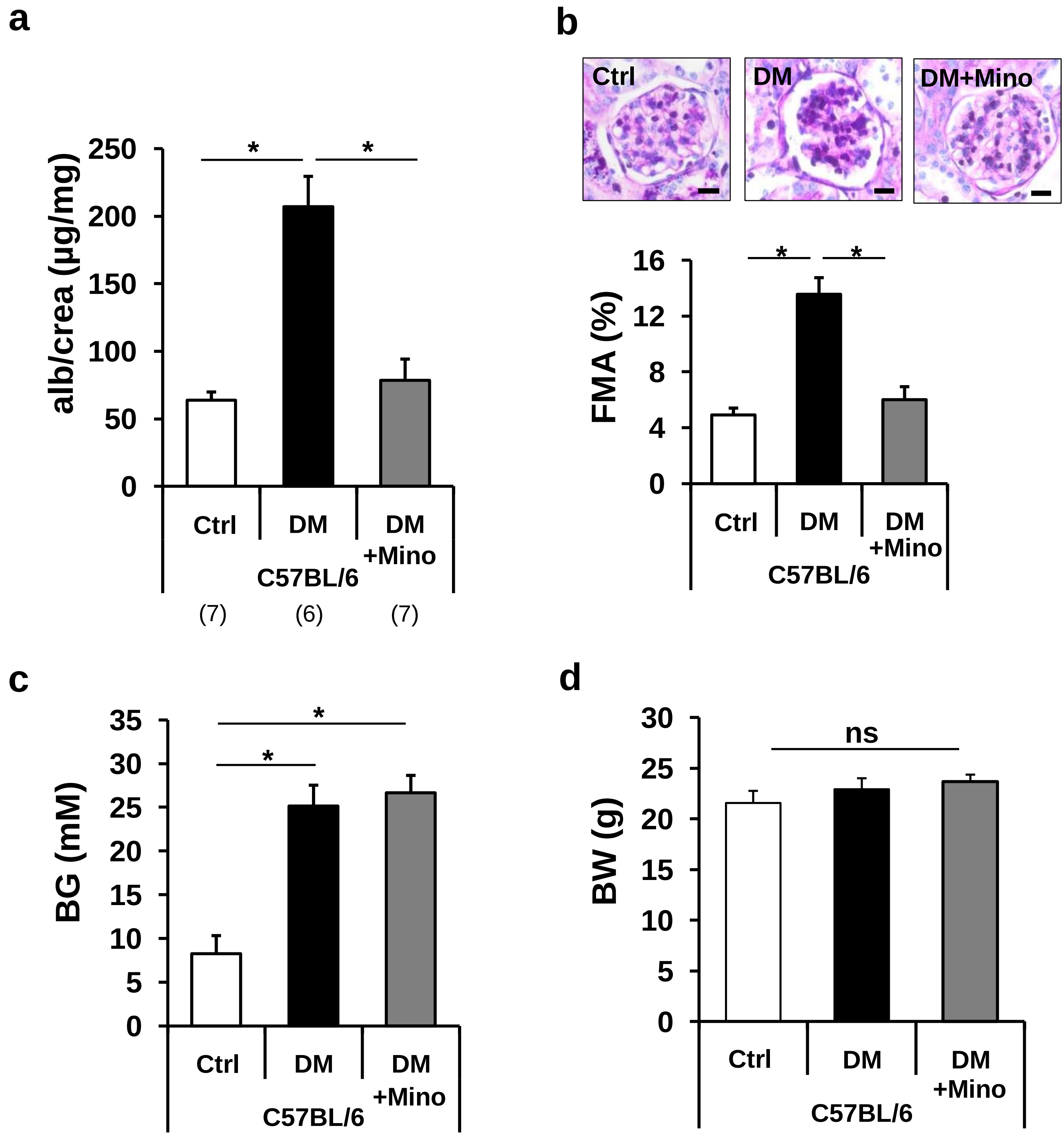
a
b
Ctrl
DM
DM+Mino
alb/crea (µg/mg)
250
200
150
100
50
0
*
*
Ctrl
DM
DM +Mino
C57BL/6
(7)
(6)
(7)
FMA (%)
16
12
8
4
0
c
BG (mM)
35
30
25
20
15
10
5
0
d
BW (g)
ns

Supp. Fig 2

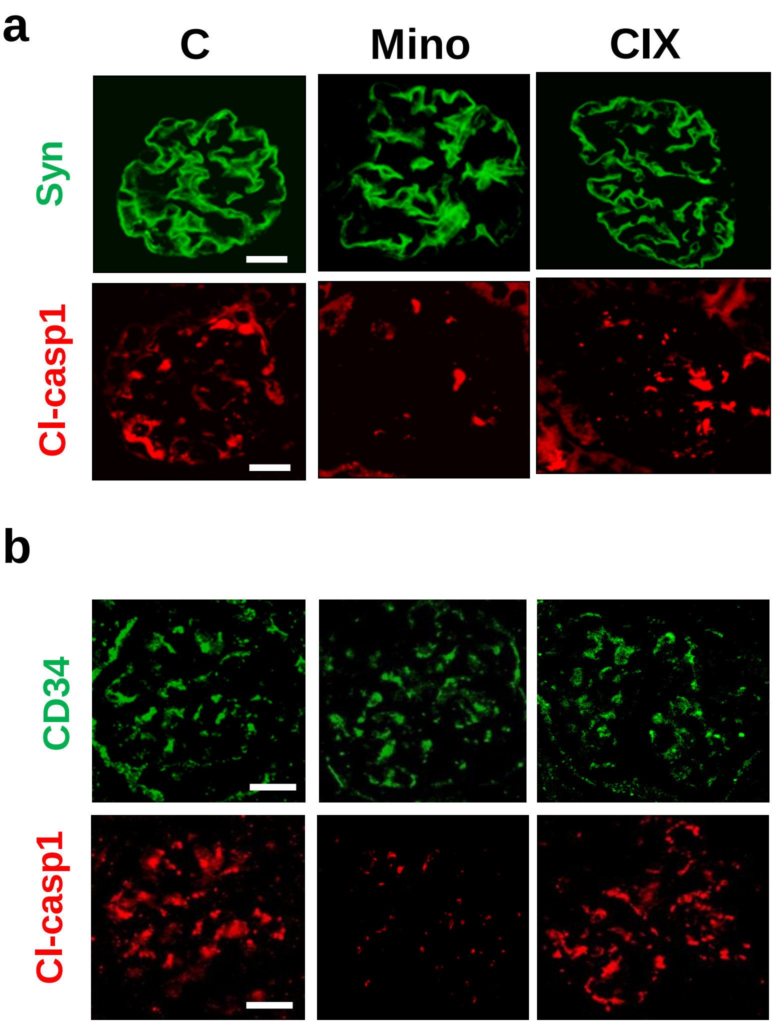

Supp. Fig 3

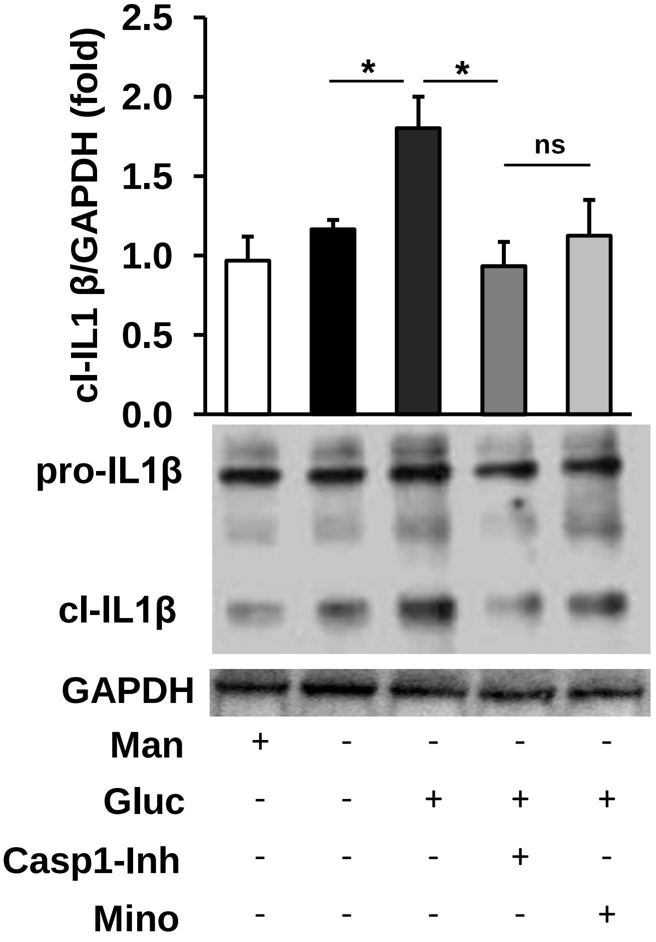

2.5
2.0
1.5
1.0
0.5
0.0
cl-IL1 β/GAPDH (fold)
*
*
ns
pro-IL1β
cl-IL1β
GAPDH
Man + - - - -
Gluc - - + + +
Casp1-Inh - - - + -
Mino - - - - +

Supp. Fig 4

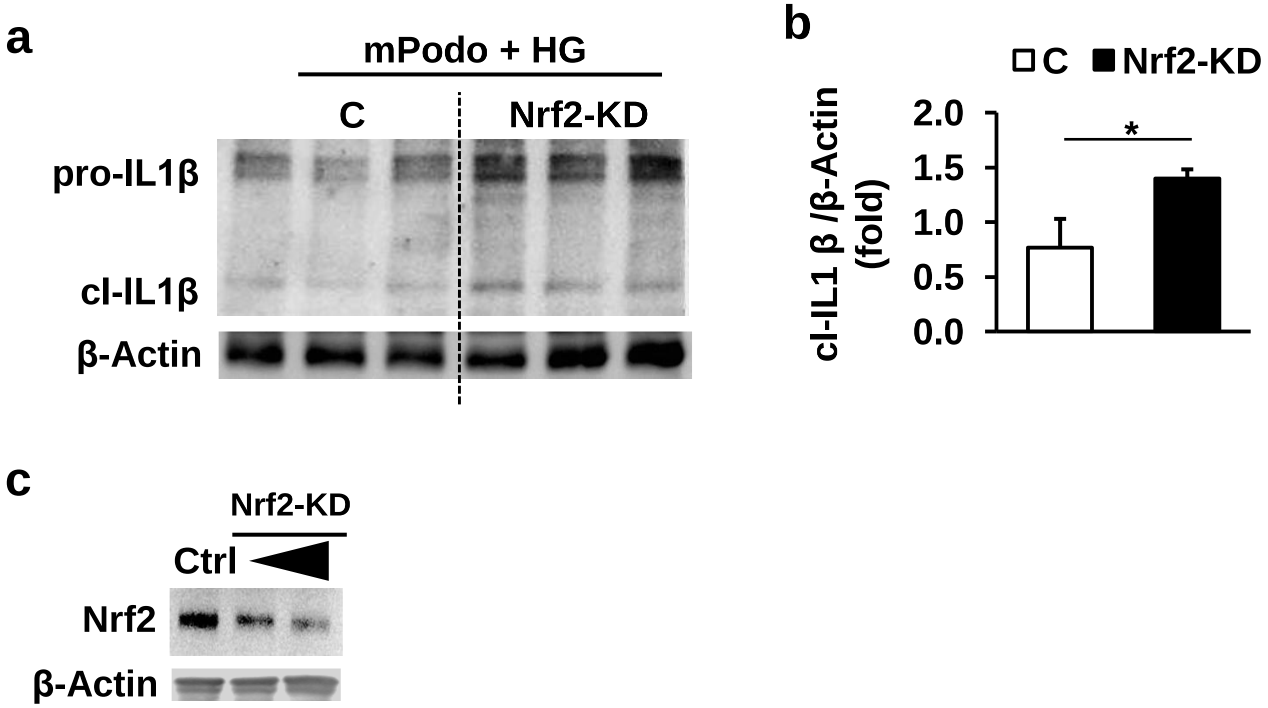
a
mPodo + HG
C
Nrf2-KD
pro-IL1β
cl-IL1β
β-Actin
b
C
Nrf2-KD
cl-IL1 β /β-Actin (fold)
2.0
1.5
1.0
0.5
0.0
*
c
Nrf2-KD
Ctrl
Nrf2
β-Actin

Supp. Fig 5

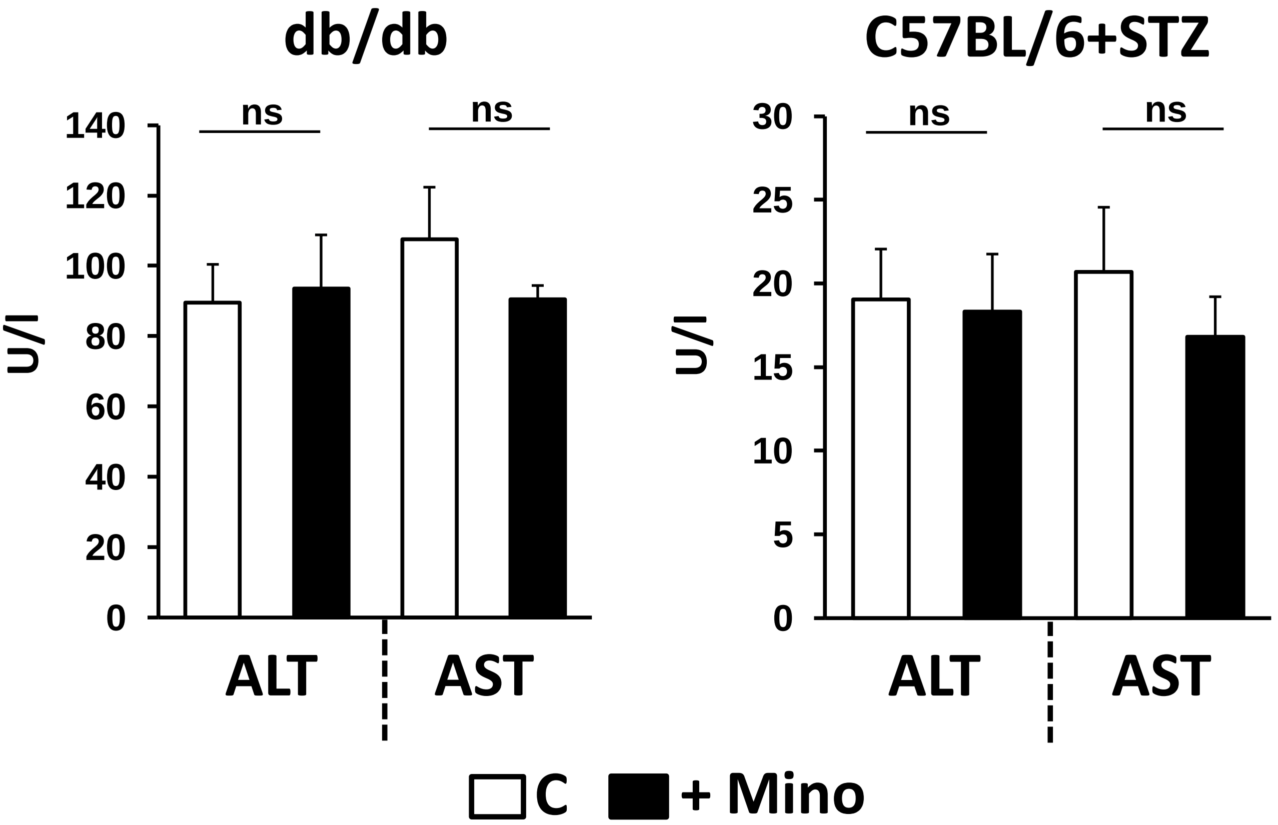

db/db
C57BL/6+STZ
ns
ns
ns
ns
140
120
100
80
60
40
20
0
30
25
20
15
10
5
0
U/l
U/l
ALT
AST
ALT
AST
C
+ Mino

Supp. Fig 6

a. for Figure 2a

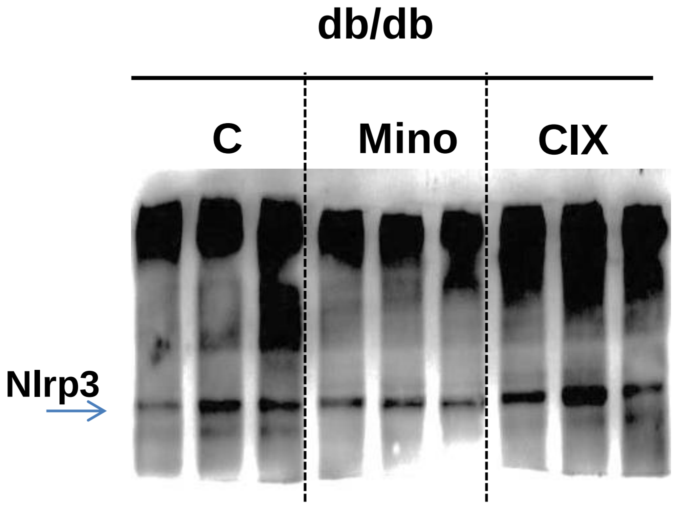

b. for Figure 3a

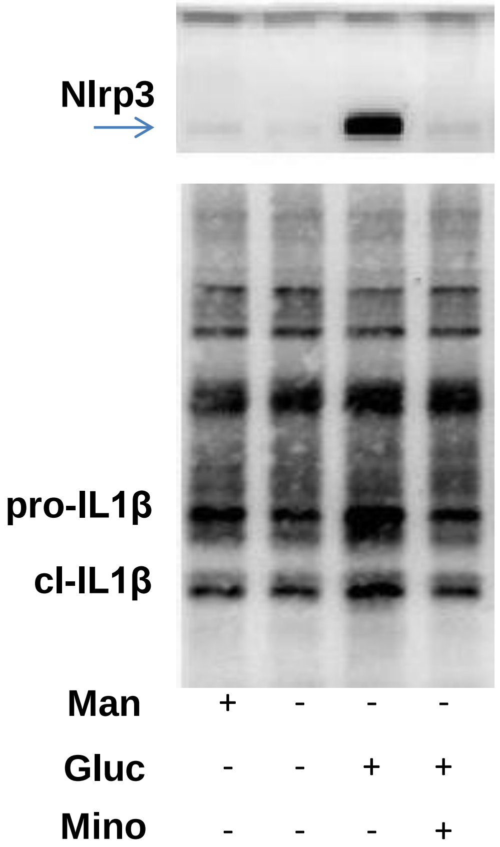

c. for Figure 3b

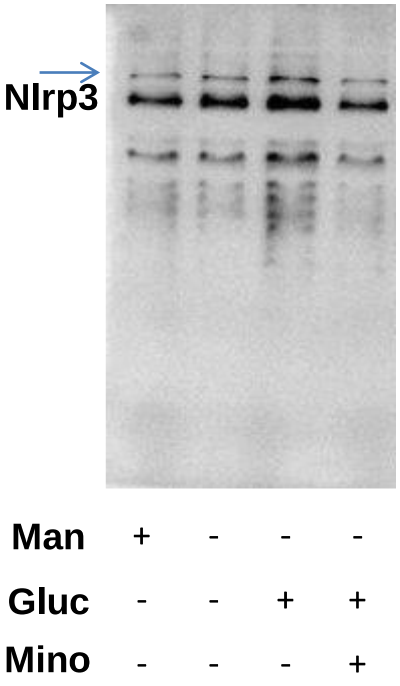

d. for Figure 3c

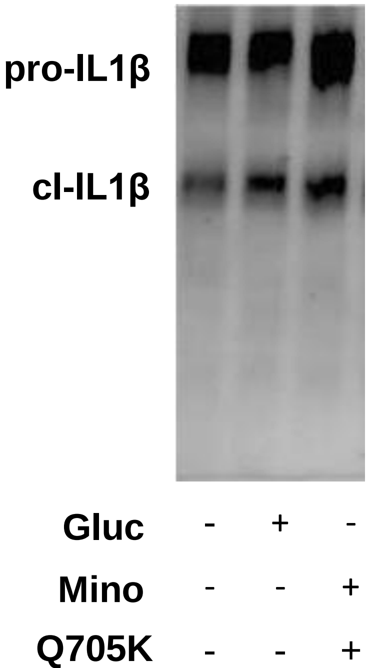

Supp. Fig 6 continued

e. for Figure 4e

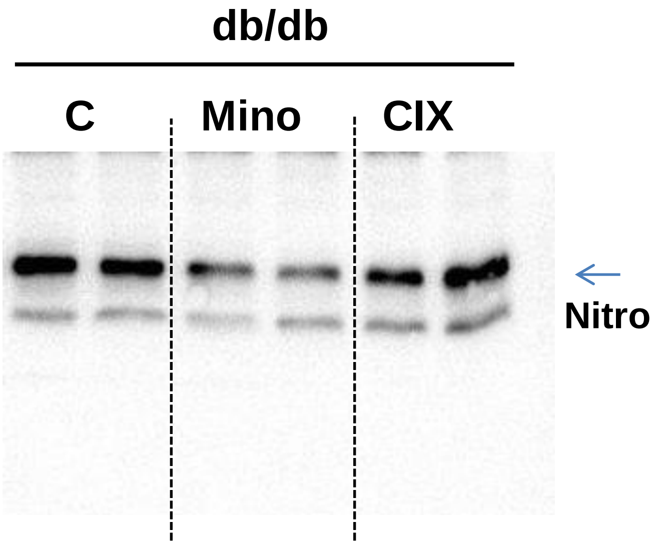

f. for Figure 4f

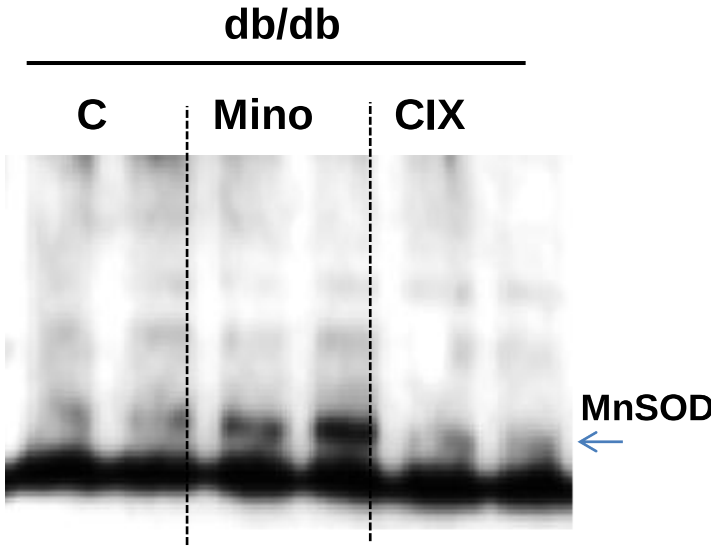

g. for Figure 5a

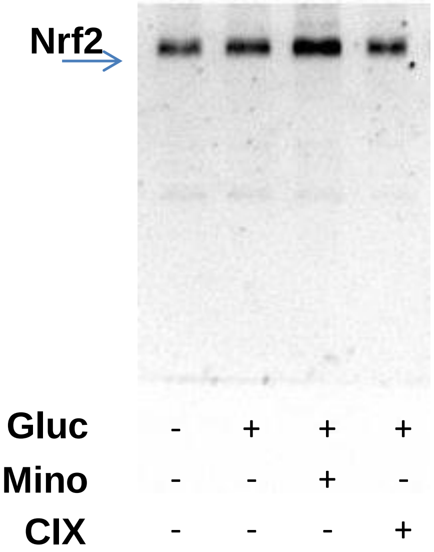

h. for Figure 5b

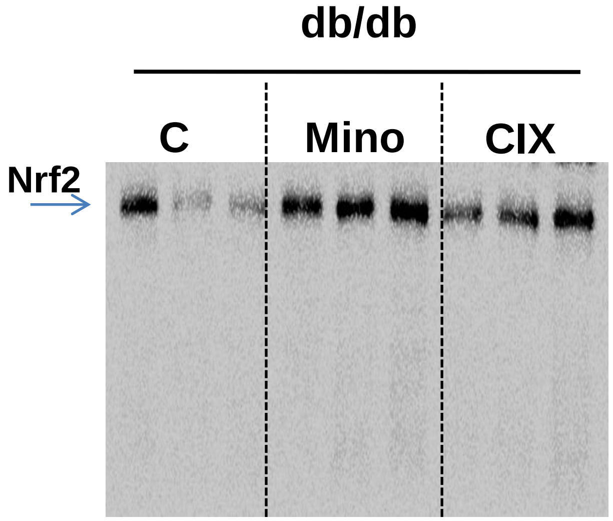

Supp. Fig 6 continued

i. for Figure 6a

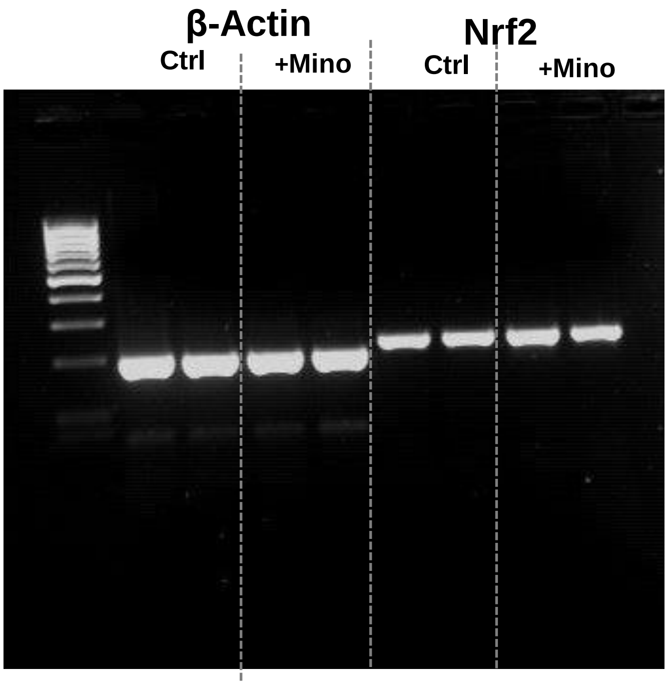

j. for Figure 7a

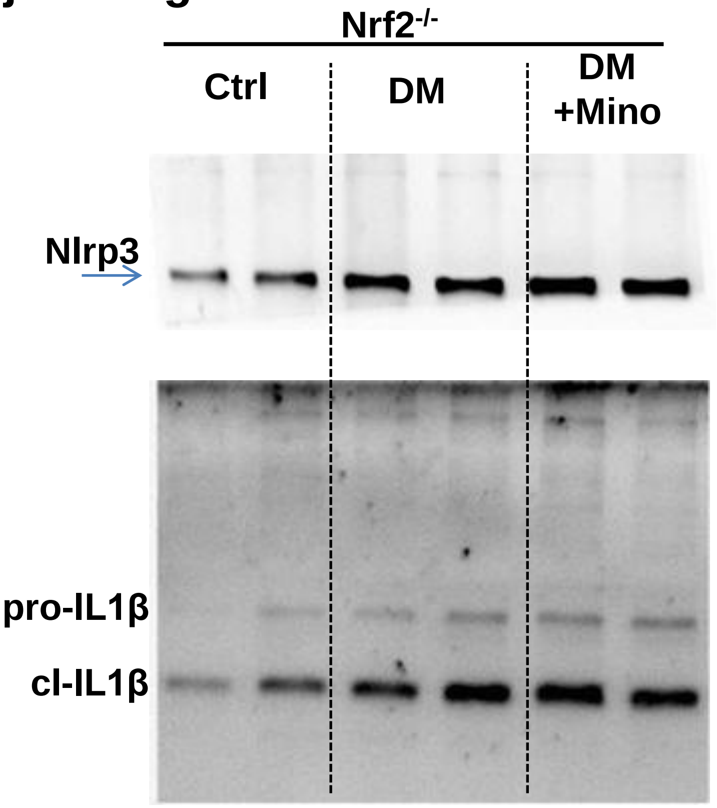

Supplement: Supplementary Information [file srep34228-s1.pdf]
